# Body and Brain Weariness

**Published:** 1897-03

**Authors:** 


### Body and Brain Weariness.

Dr. Miller tells us in the *Journal of Hygiene* that the body is wearied more quickly when the mind is tired.

"The child fatigues much more readily—that is, his organism is more quickly depleted and poisoned—during the periods of most rapid growth. The average boy has his most rapid growth between the age of fourteen and sixteen—in these two years he increases in weight by as much as he did during the entire six years preceding the age of fourteen. At this period of most rapid growth—the period of pubescence—the brain loses considerable weight, because of the fact that the usual blood supply is lessened by a portion being withdrawn to nourish the viscera and other organs undergoing rapid revolutional changes during this period. While the weight of the brain is but one forty-fifth of that of the whole body, it requires one-eighth of all the blood to nourish it.

"At no time in his whole school career is the boy more deserving of sympathy than at the time of most rapid growth. In all learning two features are involved: Proper presentation of material by the teacher, and proper attitude of mind on the part of the student. Seldom, if ever, can the latter condition be supplied by the boy or girl in the midst of the physical and mental revolutions and evolutions of pubescence.

"The great curse of this age is the demand for rapid education. Parents and teachers crowd the children through a long, hard year's work. Health is sacrificed for promotion. What is learned while a child is fatigued is soon lost, the mind's force being equally dissipated. Vital force is required faster than it is generated. The work of to-day is done on to-morrow's credit, and the system of a child is wholly at a loss to protect itself against disease and accident."

